# A simple adaptation to a protein crystallography station to facilitate difference X-ray scattering studies

**DOI:** 10.1107/S1600576719001900

**Published:** 2019-03-28

**Authors:** Amit Sharma, Peter Berntsen, Rajiv Harimoorthy, Roberto Appio, Jennie Sjöhamn, Michael Järvå, Alexander Björling, Greger Hammarin, Sebastian Westenhoff, Gisela Brändén, Richard Neutze

**Affiliations:** a Department of Chemistry and Molecular Biology, University of Gothenburg, Box 462, 40530 Gothenburg, Sweden; b Multidisciplinary Centre for Advanced Research and Studies, Jamia Millia Islamia, New Delhi 110025, India; c ARC Centre of Exellence in Advanced Molecular Imaging, Department of Chemistry and Physics, La Trobe Institute for Molecular Science, La Trobe University, Victoria 3086, Australia; d MAX IV Laboratory, Box 118, 221 00 Lund, Sweden

**Keywords:** difference X-ray scattering, protein structural changes

## Abstract

The X-ray crystallography station I911-2 at MAXLab II (Lund, Sweden) has been adapted to enable difference small- and wide-angle X-ray scattering (SAXS/WAXS) data to be recorded. Modifications to the beamline included a customized flow cell, a motorized flow cell holder, a helium cone, a beam stop, a sample stage and a sample delivery system. This setup incorporated external devices such as infrared lasers, LEDs and reaction mixers to induce conformational changes in macromolecules. This platform was evaluated through proof-of-principle experiments capturing light-induced conformational changes in phytochromes. A difference WAXS signature of conformational changes in a plant aqua­porin was also demonstrated using caged calcium.

## Abstract 2

A setup is constructed to facilitate difference X-ray scattering studies at a synchrotron-based protein crystallography beamline. This setup provides a flexible platform for preparative studies of protein structural dynamics.

## 1. Introduction   

Small- and wide-angle X-ray scattering is sensitive to changes in protein secondary structure in solution. Wide-angle X-ray scattering (WAXS) allows the collection of X-rays scattered from macromolecules in solution to higher angles than is typical in small-angle X-ray scattering (SAXS) studies of proteins. Difference WAXS has become an important method of studying protein conformational changes (Makowski, 2010 ▸; Tiede *et al.*, 2002 ▸; Hirai *et al.*, 2004 ▸; Minh & Makowski, 2013 ▸; Rodi *et al.*, 2007 ▸; Fischetti *et al.*, 2004 ▸). When used in combination with short X-ray pulses generated using synchrotron radiation (Andersson *et al.*, 2009 ▸; Malmerberg *et al.*, 2011 ▸, 2015 ▸; Ahn *et al.*, 2009 ▸; Cammarata *et al.*, 2008 ▸; Cho *et al.*, 2010 ▸; Berntsson *et al.*, 2017 ▸; Takala *et al.*, 2014 ▸; Ramachandran *et al.*, 2011 ▸) or X-ray free-electron lasers (Arnlund *et al.*, 2014 ▸; Levantino *et al.*, 2015 ▸), time-resolved WAXS (TR-WAXS) data can be collected in order to follow protein structural changes on timescales from picoseconds to seconds.

TR-WAXS relies upon the accurate measurement of small changes in X-ray scattering intensities due to the build-up of transient populations of reaction intermediates. All TR-WAXS studies reported to date have examined conformational changes in light-active proteins such as carbon monoxide bound to the active site of globin proteins (Ahn *et al.*, 2009 ▸; Levantino *et al.*, 2015 ▸; Cammarata *et al.*, 2008 ▸; Cho *et al.*, 2010 ▸), rhodopsins (Andersson *et al.*, 2009 ▸; Malmerberg *et al.*, 2011 ▸, 2015 ▸), photoactive yellow protein (Ramachandran *et al.*, 2011 ▸), photoreceptors (Berntsson *et al.*, 2017 ▸; Takala *et al.*, 2014 ▸) and photosynthetic reaction centres (Arnlund *et al.*, 2014 ▸). When using light as a trigger, TR-WAXS data can be recorded from both the reference (dark) and the photo-activated (light) states of the same sample. This allows difference signals to be calculated where systematic experimental uncertainties cancel, for example, background scatter, detector background and variations in protein concentration. For these reasons, even though X-ray scattering differences are very small (typically of the order of 0.1% or less of the scattering intensity from the solvent in which the protein is immersed) they can be measured accurately. A related emerging method is time-resolved serial crystallography (Nango *et al.*, 2016 ▸; Suga *et al.*, 2017 ▸; Pande *et al.*, 2016 ▸; Tenboer *et al.*, 2014 ▸; Barends *et al.*, 2015 ▸; Coquelle *et al.*, 2018 ▸; Nogly *et al.*, 2018 ▸), which allows time-resolved difference electron density maps to be recorded at X-ray free-electron lasers (XFELs). This approach is expanding the scope of time-resolved crystallography and also points towards a growing scientific interest in the field of time-resolved structural biology (Neutze & Moffat, 2012 ▸).

It is not possible to pursue TR-WAXS studies of protein structural dynamics without access to synchrotron or XFEL radiation. A significant limitation for the field has therefore been the availability of access to specialized time-resolved beamlines such as ID09B at the ESRF and BioCARS at the APS, with the consequence that projects often must be at a mature stage before beam time is granted. Access to XFEL user facilities such as the LCLS, SACLA, SwissFEL and the EU-XFEL is even more competitive. As such, further growth of the field is hindered by the ‘chicken-and-egg problem’, where a pressing need from the scientific community to address a much broader class of biological problems is in conflict with the fact that projects at a very early stage are not competitive for beam time, or the investment required for even simple test experiments is too high.

To address this issue, we present here a simple adaptation to a protein crystallography beamline that facilitates preparative studies of difference WAXS without the need for access to highly specialized beamlines. This setup can be installed and dismounted within a single shift of beam time and adds optional functionality to the most widely used class of beamlines in the synchrotron radiation community. Availability of such an option would allow scientists access to preparative difference X-ray scattering experiments where they could test reaction initiation conditions and sample environments in order to ensure the feasibility of their scientific objective, and gain valuable preliminary data to support applications for more specialized instruments. The low overhead of this approach allows for the possibility of multiple failures as the experimental design is optimized. Aspects of the platform could be adapted to allow serial crystallography studies at existing protein crystallography beamlines if used in combination with an appropriate sample delivery system (Weierstall *et al.*, 2014 ▸, 2012 ▸; Fuller *et al.*, 2017 ▸), and some aspects of the sample stage and control systems could also be applied at specialized SAXS stations.

## 2. Methods   

### 2.1. Beamline setup   

**Figure 1:**
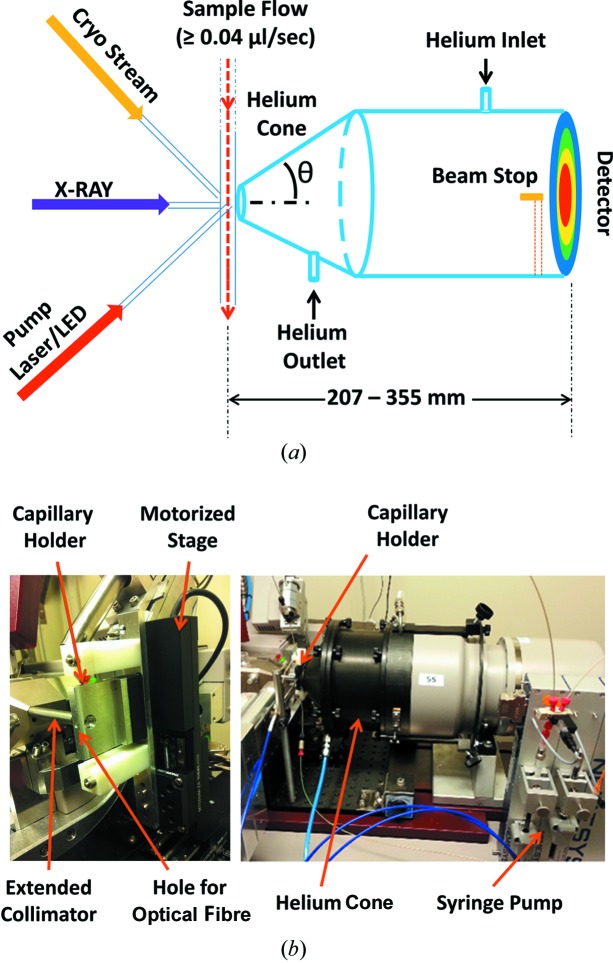
Difference SAXS/WAXS setup built on the protein crystallography beamline I911-2 of MAXLab. (*a*) Schematic of the setup, showing the sample flow through a capillary held in a cryo-stream environment. The sample is activated using a laser or LED and X-rays are used as the structural probe. X-rays scattered from the sample pass through the helium cone and are collected on the detector. X-ray scattering angles can be varied by moving the detector relative to the sample position. The direct beam is blocked by a beam stop placed close to the detector. (*b*) Photographs of the experimental setup. The left panel shows the capillary holder held by a motorized stage and the path of the X-ray beam, delivered onto the sample using an extended collimator. A hole drilled into the side of the capillary holder was used to hold an optical fibre and thereby illuminate the region of the capillary exposed to X-rays. The right panel shows the setup on the beamline during these experiments.

This work was performed at the I911-2 3.5 T super-conducting wiggler beamline (Ursby *et al.*, 2004 ▸) with a photon flux of 10^11^ photons s^−1^ and an X-ray wavelength 1.04 Å. This station is a dedicated macromolecular crystallography beamline equipped with a MARCCD 2048 square pixel detector. A steady-state WAXS setup (Fig. 1) was constructed on this beamline, which facilitated the accurate measurement of difference SAXS/WAXS signals from proteins. X-ray scattering data were collected over the *q* range from 0.06 to 2.2 Å^−1^ by moving the detector as close as 207 mm from the sample or by taking it as far back as 355 mm. The characteristic features of the design are summarized in Table. 1 ▸. The setup was developed around four major components: (i) the sample delivery system; (ii) a custom-built helium cone; (iii) externally coupled devices; and (iv) the software control.

### 2.2. Sample delivery system   

Protein samples were pumped through a quartz capillary (1.0 mm in diameter and 10 µm wall thickness) attached using heat-shrink seals to PEEK (polyether ether ketone) tubing (1/16 inches with an internal diameter of 0.75 inches; Idex Health & Science). This tubing was connected to a neMESYS Low Pressure Syringe Pump (Fig. 1 ▸) which was run with a stable non-pulsating flow rate ≥ 0.04 µl s^−1^. The capillary was mounted in a custom-made capillary holder [Fig. 1 ▸(*b*)] aligned in the X-ray beam with the help of a motorized *xyz* stage (Thorlabs). Since the sample was mounted behind the protein crystallography goniometer, an extension to the collimator was placed after the usual X-ray beam exit position but before the quartz capillary. This extension of the collimator was essential to create the space surrounding the sample position for these studies as the local environment of the protein crystallography goniometer was quite specialized and crowded.

### 2.3. Custom-built helium cone   

**Figure 2:**
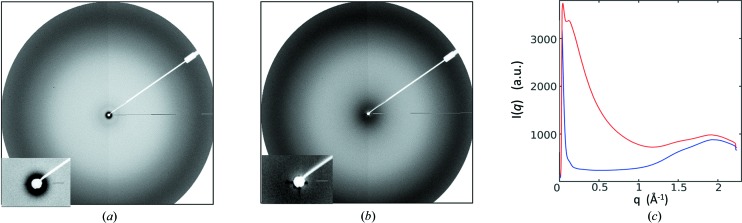
Comparison of the effect of the helium cone on total X-ray scattering. (*a*) X-ray scattering when the helium cone is filled with helium. (*b*) X-ray scattering when the helium cone is filled with air. (*c*) X-ray scattering after azimuthal integration from phytochrome samples before (red) and after (blue) helium was passed through the cone.

X-ray scattering signals at all angles are affected by background from air scattering. Ideally, the beam path between the sample and the detector is enclosed under vacuum (Dubuisson *et al.*, 1997 ▸; Blanchet *et al.*, 2012 ▸), as is standard on dedicated SAXS stations. Since vacuum systems involve additional complexity, we opted instead to use a helium cone [Fig. 1 ▸(*b*)]. This was made of aluminium and consisted of three parts: (i) a detector attachment, (ii) an elongation tube (or tubes) and (iii) a cylindrical cone. All shared a common diameter of 180 mm and were clamped together as required. The detector attachment section was 42 mm long. It was machined to slip snugly around the circular MARCCD detector with an overlap of 16 mm and contained an O-ring to prevent excessive helium leakage. This section also included a helium gas inlet on the top and an outlet at the bottom, as well as a custom-built beam stop close to the detector. For these studies, the beam was aligned manually, but a better design would use an *xy* stage associated with the X-ray detector. Once the beam-stop alignment was satisfactory, up to three additional helium cone extensions (50, 100 and 200 mm in length) could be selected or combined to achieve the required helium cone length. Finally, a circularly truncated cone, 67 mm in length, is tapered down to the inlet where the X-ray beam passes through the helium cone. The entrance into the helium cone was covered with a silicon nitride membrane (membrane size: 8.0 × 8.0 mm; thickness: 500 nm; Silson Ltd, England). When helium was passed through the cone [Fig. 2 ▸(*a*)], the X-ray scattering was drastically reduced compared with when the cone is filled with air [Fig. 2 ▸(*b*)]. The effect of helium in lowering the background is clearly visible in Fig. 2 ▸(*c*), where plots in red and blue are the respective curves recovered after ring integration of the X-ray scattering data before and after helium was passed through the cone.

### 2.4. Externally coupled devices   

A one-to-two fan-out multimode optical fibre cable (Thorlabs, 800 µm-diameter fibre) with SMA905 connectors on all three ends was used to couple two different light sources onto the sample position. Thorlabs fibre-coupled LEDs with SMA905 fibre connectors were used to illuminate samples at the chosen visible and UV wavelengths. A flow cell was designed with an inlet for the optical fibre drilled to fit an SMA905 connector [Fig. 1 ▸(*b*)]. This inlet was placed such that the fibre tip almost touched an X-ray transparent capillary through which the sample passed and that was held within a channel drilled for this purpose. Light from the optical fibre was spread out slightly and the X-ray beam was 300 × 300 µm. Therefore, the sample volume exposed to X-rays was completely immersed in light.

**Figure 3:**
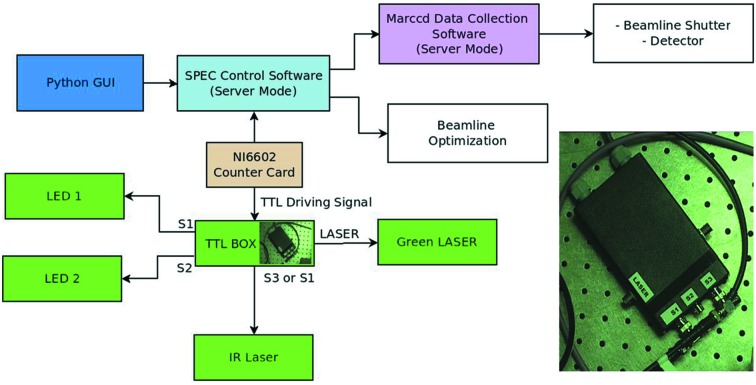
Schematic of the driving system for the setup. A Python-based graphical user interface (Python GUI) was used to operate the Unix-based software package *SPEC*, which was used in turn to control different components of the system. This schematic illustrates the following components: (i) the excitation sources (LEDs and lasers); (ii) the *MARCCD* data collection software that controls the detector and the beamline shutter; and (iii) the beamline optimization system. The schematic also shows the TTL generator connected to the digital counter card (NI6602 card) that helps drive various excitation sources. The TTL generator box is shown in the inset.

To enable the collection of WAXS data from the sample both before and after the conformational change takes place, the excitation sources were controlled using a transistor–transistor logic (TTL) generator. Fig. 3 ▸ is a block diagram showing the working principle of the experiment, where outputs S1, S2 and S3 from the TTL box drive lasers or other equipment. X-ray scattering differences during heating of the solvent, which can be due to energy being deposited in the sample by the diode or laser excitation sources, were measured using heating induced by a continuous wave infrared (CWIR) diode laser. Light- and IR-induced heating results in a strong difference X-ray scattering signal near *q* ≃ 2.0 Å^−1^, but a heat-induced difference signal can also contain changes in X-ray scattering in the low-*q* region that must be separated from those due to protein structural changes. The heating-induced component of difference scattering measurements (*e.g.* laser on – laser off) can be isolated by reproducing this heating effect on the X-ray scattering using the CWIR laser (1470 nm), which causes heating without inducing a protein structural change.

The experiment was carried out using a cycle of three images per sequence collected in series in the following order: all signals low (laser off and IR laser off), laser signal high (laser on and IR laser off) and then IR signal high (laser off and IR laser on). In the case of our studies of phytochromes, laser on corresponded to blue-light excitation and laser off corresponded to red-light illumination, the latter of which returned the phytochrome to its resting conformation.

### 2.5. Software control   

The beamline optics were controlled through the Unix-based software package *SPEC* (Certified Scientific Software). The *MARCCD* software was used as an interface to the detector. The sample-to-detector distance was calibrated by collecting X-ray scattering from lanthanum hexaboride (LaB_6_) and the powder diffraction was analysed using *Fit2D* (Hammersley *et al.*, 1994 ▸; Hammersley, 2016 ▸).

A user interface was developed using a QT-python program coupled to a TTL generator located inside the experimental hutch (Fig. 3 ▸) and connected to the digital input/output counter card (NI6602 card) to drive the various excitation sources connected to it. Up to four devices could be connected and each signal (S1 to S4) was set as high level (5 V) or low level (0 V) via a command from the control software. The sequence of high/low level for each output signal was defined depending on the devices being controlled and the sequence in which it was desired to operate them.

### 2.6. Data processing   

Azimuthal integration using the program *BioXTAS RAW* (Nielsen *et al.* 2009 ▸; Hopkins *et al.*, 2017 ▸) converted the two-dimensional scattering images collected on the MARCCD detector into one-dimensional plots showing the variation in scattered intensity with the scattering vector magnitude (*q* = 4π sin θ/λ, where λ is the X-ray wavelength and θ is half the angle by which the X-rays are deflected). X-ray scattering curves were normalized near one of the water-heating isosbestic points of 2.1 ≤ *q* ≤ 2.2 Å^−1^, at which the X-ray scattering during heating of the sample is unchanged. Statistical data rejection criteria were implemented (Andersson *et al.*, 2009 ▸; Arnlund *et al.*, 2014 ▸; Malmerberg *et al.*, 2011 ▸, 2015 ▸) to remove outliers which may arise from variations in the beam intensity or other experimental errors, such as the appearance of air bubbles, back pressure, and variations in protein concentration or viscosity. These outlier rejection criteria include (i) normalizing the scattering curves over the domain 2.1 ≤ *q* ≤ 2.2 Å^−1^; (ii) discarding curves that retained intensities above a cap of 0.8 in the region 1.0 ≤ *q* ≤ 1.8 Å^−1^ after normalization (this removed extreme outliers such as curves with air bubbles or detector readout errors *etc*.); and (iii) averaging all remaining curves and rejecting those which deviated by more than 0.8 standard deviations from the average over the set for the domain 0.2 ≤ *q* ≤ 2.1 Å^−1^.

### 2.7. Sample preparations   

#### 2.7.1. Phytochromes   

Photosensory core module (PAS-GAF-PHY) phytochrome was purified from *Deinococcus radiodurans* as previously described (Takala *et al.*, 2014 ▸). In brief, the His-tagged protein in 30 m*M* Tris–HCl, 150 m*M* NaCl pH 8.0, was produced in *Escherichia coli (*BL21 DE3 cells) and purified by Ni^2+^-affinity and size-exclusion chromatography.

#### 2.7.2. SoPIP2;1   

Wild-type spinach aqua­porin (SoPIP2;1) was overexpressed in the methyl­otropic yeast *Pichia pastoris* as previously described (Törnroth-Horsefield *et al.*, 2006 ▸). In brief, this membrane protein was suspended in the detergent *n*-octyl-β-d-glucoside and ultracentrifugation, cation-exchange chromatography and gel-filtration steps were used to obtain a pure sample. A second gel filtration was performed just before the experiment to remove aggregates. The final working sample was equilibrated in 20 m*M* Tris–HCl, 100 m*M* NaCl pH 7.5, and 1% *n*-octyl-β-d-glucoside was used at 15 mg ml^−1^ (Table 2 ▸).

## 3. Results   

### 3.1. Photoinduced conformational changes in phytochromes   

We conducted a proof-of-principle study to reproduce light-induced structural changes in bacteriophytochrome from *D. radiodurans.* Phytochromes can be reversibly driven between two states Pr and Pfr, using two coloured illuminations, yielding a difference SAXS/WAXS signal (Berntsson *et al.*, 2017 ▸; Björling *et al.*, 2015 ▸; Takala *et al.*, 2014 ▸). Thus, this system provides an ideal model system to conduct light-induced conformational studies using WAXS.

**Figure 4:**
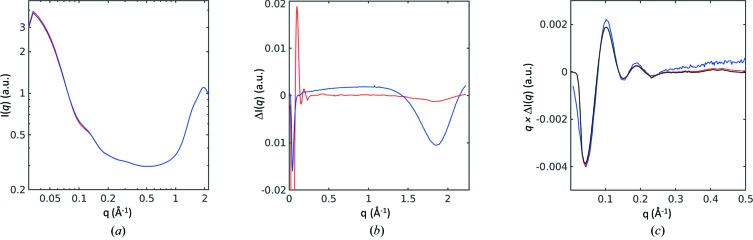
Light-induced changes in WAXS data collected from phytochromes. (*a*) log[*I*(*q*)] versus log(*q*) SAXS/WAXS scattering for phytochromes. The red line was recorded after samples were illuminated with 780 nm light whereas the blue line was recorded after samples were illuminated with 660 nm light. The plot shows data collected for 16 000 s at the rate of 12 s exposure per frame. The influence of the beam stop is seen as a sharp drop in the X-ray scattering intensity at very low *q*. (*b*) Difference WAXS scattering signal recovered from phytochromes (red line): Δ*I*(*q*) = *I*(*q*)^Pfr^ − *I*(*q*)^Pr^, after samples were illuminated with 780 or 660 nm light. The difference WAXS scattering due to heating (blue line): Δ*I*(*q*) =  I(*q*)^IR^ − *I*(*q*)^dark^, where IR light of 1470 nm was used to heat the samples without initiating the light-driven conformational change. (*c*) Difference WAXS signal *q*Δ*I*(*q*) showing data recorded from phytochromes before (red) and after (black) the influence of heating was removed from the signal. For comparison, similar data that were recorded at the cSAXS beamline of the SLS are shown (blue).

Two LED sources emitting light at 780 and 660 nm and operating at 13 mW were used to switch the sample from Pfr to Pr and Pr to Pfr, respectively. A total volume of 400 µl of sample at 14 mg ml^−1^ was circulated (*i.e.* pumped in both directions, reversing every 300 µl) through the 1 mm-diameter capillary at a flow rate of 0.5 µl s^−1^. The sample was exposed to 780 or 660 nm light from either LED as it flowed through the X-ray beam position, with the X-ray detector read out after 12 s exposure. The applied light fluence ensured a rapid interchange between the two states. Approximately 1300 frames were collected following exposure with 780 nm light (red line) and 660 nm light, with alternate wavelength illumination toggled by the software control. Data were collected using an X-ray exposure of 12 s per frame. Fig. 4 ▸(*a*) shows the X-ray scattering profiles after azimuthal integration, recorded following exposure to both 780 nm (red line) and 660 nm light (blue line). The *I*(*q*) versus *q* plot shows that the quality of the X-ray scattering measurements is acceptable over the full measurement domain.

Difference WAXS curves between the two LED exposures were calculated, *I*(*q*)^Pfr^ − *I*(*q*)^Pr^ [red line, Fig. 4 ▸(*b*)], and showed the change in the SAXS (*q* ≤ 0.3 Å^−1^) region that is characteristic of the protein conformational change (Berntsson *et al.*, 2017 ▸; Björling *et al.*, 2015 ▸; Takala *et al.*, 2014 ▸). Moreover, a change in X-ray scattering in the WAXS region (1.4 ≤ *q* ≤ 2.2 Å^−1^) that is associated with light-induced sample heating due to the two diodes, causing the sample to heat to slightly different temperatures, was also observed. Illumination with an IR laser (1470 nm) was used to induce sample heating but without photo-activating the phytochromes. Comparison of the X-ray scattering curves for IR laser on versus IR laser off reveals the light-induced dip in the WAXS region [1.4 ≤ *q* ≤ 2.2 Å^−1^, blue line, Fig. 4 ▸(*b*)] but with a relatively small effect on the SAXS region (*q* ≤ 0.3 Å^−1^) (Makowski, 2010 ▸). The influence of light-induced heating was removed from the recorded scattering [red line, Fig. 4 ▸(*c*)] by scaling the IR-induced heating curve [blue line, Fig. 4 ▸(*b*)] to match the (smaller) heating signature at high *q*, associated with photoactivation of the samples [red line, Fig. 4 ▸(*b*)]. This correctly scaled heating curve was then subtracted from the photoactivated difference scattering curve to recover the pure light-induced (*i.e.* heating removed) changes in X-ray scattering [black line, Fig. 4 ▸(*c*)]. In this particular example, this heat correction had very little effect, but in other studies it may be more significant. It is important to be able to make these control measurements during the data collection sequence and have all data collected from the same sample, since even very small differences in protein concentration can lead to differences in X-ray scattering that are of the order of the light-induced changes in X-ray scattering. This is an important consideration when developing any TR-WAXS project and it is valuable to be able to make these distinctions with confidence at an early stage of a project.

Finally, the experimental changes measured in the SAXS domain [black line, Fig. 4 ▸(*c*)] were compared with previously published difference SAXS measurements from the same protein (Takala *et al.*, 2014 ▸) made at the cSAXS beamline of the Swiss Light Source (SLS) [blue line, Fig. 4 ▸(*c*)]. It is apparent that these measurements are almost identical for *q* ≤ 0.25 Å^−1^, yet the signal-to-noise ratio associated with the MAXLab data appears modestly better at higher X-ray scattering angles. In this comparison, the total experimental time used at MAXLab (16 000 s of data collection with a photon flux of 10^11^ photons s^−1^) was an order of magnitude longer than that used to collect data at the SLS (1644 s of data collection with a photon flux of 10^12^ photons s^−1^) and this compensated for the lower flux available from the MAXLab synchrotron. Nevertheless, it is encouraging that this adaption of a standard protein crystallography beamline yielded similar quality data for the difference X-ray scattering signal when the total X-ray fluence through the sample was similar.

### 3.2. Structural changes induced in SoPIP2;1 using caged compounds   

As an example of preliminary experiments on a system from which no TR-WAXS data have been reported, we used the same setup to study biochemically induced structural changes in a gated plant aqua­porin. SoPIP2;1 is known to regulate water transport across the plant plasma membrane, and X-ray crystallography studies have revealed two conformations in which the open or closed state of the water channel is controlled by the conformation of cytoplasmic loop D (Törnroth-Horsefield *et al.*, 2006 ▸). Both the crystallographic structure and functional assays (Alleva *et al.*, 2006 ▸) have suggested that calcium is one of the biochemical signals that controls the conformation of loop D and therefore controls the functionality of the aqua­porin.

Caged calcium was used as a biochemical trigger that could be released by photoactivation (Kaplan & Somlyo, 1989 ▸; Ellis-Davies *et al.*, 1996 ▸). DM-Nitro­phen was used as a commercially available source of caged calcium that is released into solution upon exposure to UV light. Conditions required to fully release Ca^2+^ using a 365 nm diode coupled to the sample through an optical fibre were determined using the absorption change of the Ca^2+^-sensitive dye arsenazo III at 652 nm. Spectral data, recorded using the same flow-cell arrangement used for the WAXS studies but placed within a microspectrophotometer (Hadfield & Hajdu, 1993 ▸), established the illumination intensity and sample flow rates to be applied at synchrotron-based experiments (Järvå, 2015 ▸).

Protein solutions at a concentration of 15 mg ml^−1^ were kept on ice until they were loaded into the sample delivery system. The protein sample (300 µl) was flowed through the 1 mm capillary at 0.05 µl s^−1^ and WAXS data were collected using UV–dark data collection, where a UV diode (365 nm, Thorlabs) was used to release caged Ca^2+^. A cryostream was used to maintain the sample at 283 K at the position where the X-rays and UV light illuminated the sample. Data were collected with an X-ray exposure of 30 s and the final difference WAXS data were averaged from approximately 250 repeats [*I*(*q*)^UVon^ − *I*(*q*)^dark^]. As with the above studies on phytochromes, an IR laser (1470 nm) was used to characterize the influence of heating alone, but in this case the heating signal due to UV illumination was so weak (presumably because of the use of a cryostream cooling the sample to 283 K) that it was not necessary to remove the heating signal numerically.

**Figure 5:**
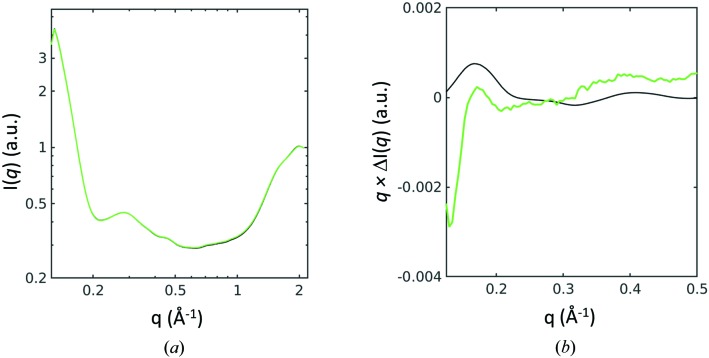
Difference WAXS changes in SoPIP2;1 associated with the release of caged Ca^2+^. (*a*) SAXS/WAXS absolute scattering before (black line) and after (green line) the sample was illuminated with UV, plotted as log[*I*(*q*)] versus log(*q*). (*b*) Experimental difference data *q*Δ*I*(*q*), where Δ*I*(*q*) = *I*(*q*)^UV^ − *I*(*q*)^dark^ is shown in green. Data were collected for 7500 s with an X-ray exposure of 30 s per frame. A prediction (black line) based on known crystallographic structures (Törnroth-Horsefield *et al.*, 2006 ▸) of the open and closed forms of SoPIP2;1 is shown for comparison.

Total X-ray scattering measurements from samples of SoPIP2;1 are shown in Fig. 5 ▸(*a*). After the UV-induced release of Ca^2+^, the resulting difference X-ray scattering signal (Ca^2+^ released minus no Ca^2+^ release, or UV on minus dark) is shown in Fig. 5 ▸(*b*) (green). This difference SAXS spectrum has the hallmarks of the expected protein structural changes since a negative difference SAXS feature is present at low resolution (*q* ≤ 0.15 Å^−1^), a positive peak is visible in the range 0.15 ≤ *q* ≤ 0.2 Å^−1^ and a second positive peak is visible at higher scattering angle, 0.3 ≤ *q* ≤ 0.45 Å^−1^. Moreover, the positions of the two positive difference SAXS/WAXS peaks show qualitative agreement with theoretical predictions, assuming that loop D of SoPIP2;1 moves from an unstructured conformation to that observed in the closed crystal structure in the presence of Ca^2+^ (Törnroth-Horsefield *et al.*, 2006 ▸). This simple prediction, however, is not in perfect agreement with the experimentally observed changes in X-ray scattering; hence additional experimental data at a specialist SAXS/WAXS station as well as a deeper theoretical modelling of conformational changes and studies of the influence of calcium on the protein detergent micelle would be required to draw strong structural conclusions. It is also possible that the release of the cage is inducing other effects such as partial protein aggregation or that the UV light is affecting the protein directly. As such, these preliminary data cannot distinguish between multiple possible models at this point but nevertheless provide an indication that reliable difference WAXS signals can be recorded and show promise for the future feasibility of this project. This is precisely the type of indicative structural information we aimed to recover by modifying a protein crystallography beamline in this manner.

## 4. Conclusions   

We developed an X-ray scattering setup on the dedicated protein crystallography beamline, I911-2, at MAXLab. This setup facilitated validation studies of known structural changes in light-sensing phytochromes and exploratory studies of putative calcium-induced structural changes in the gated aqua­porin SoPIP2;1. The difference WAXS signals were consistent with what is known about both protein systems and the signal-to-noise ratio was competitive with similar studies at more specialized beamlines [Fig. 2 ▸(*c*)]. Once established, this setup could be assembled and disassembled during a single shift.

We believe that the growing interest in time-resolved WAXS (Ahn *et al.*, 2009 ▸; Levantino *et al.*, 2015 ▸; Cammarata *et al.*, 2008 ▸; Cho *et al.*, 2010 ▸; Andersson *et al.*, 2009 ▸; Malmerberg *et al.*, 2011 ▸, 2015 ▸; Ramachandran *et al.*, 2011 ▸; Berntsson *et al.*, 2017 ▸; Takala *et al.*, 2014 ▸; Arnlund *et al.*, 2014 ▸) and time-resolved serial femtosecond crystallography (Nango *et al.*, 2016 ▸; Suga *et al.*, 2017 ▸; Pande *et al.*, 2016 ▸; Tenboer *et al.*, 2014 ▸; Barends *et al.*, 2015 ▸; Coquelle *et al.*, 2018 ▸; Nogly *et al.*, 2018 ▸) creates a need for synchrotron-based facilities at which the experimental feasibility of time-resolved studies can be explored without the need to gain access to highly specialized synchrotron- or XFEL-based experimental stations. Moreover, with rapid-readout pixel-based X-ray detectors, it is possible to achieve a time resolution of the order of milliseconds in time-resolved X-ray scattering studies (Westenhoff *et al.*, 2010 ▸). Since the overall efficiency of protein crystallography data collection has improved dramatically over the past decade, it is possible that protein crystallography stations will have time available within their user schedule for more exploratory, protocol validation experiments. We suggest that a similar setup to that described here could provide a viable route that would allow users of other synchrotron radiation facilities to explore and optimize experimental conditions for reaction initiation. Moreover, this flexible setup, including the beamline pipe-extension, sample stage, helium cone and control routines, could also allow a protein crystallography station to be adapted for exploratory serial millisecond crystallography studies (Nogly *et al.*, 2015 ▸) when using an appropriate sample delivery platform (Weierstall *et al.*, 2014 ▸, 2012 ▸; Fuller *et al.*, 2017 ▸). This level of flexibility will be valuable in supporting the increasing user interest that is driving the growth of the field of time-resolved studies of protein reaction dynamics.

## Figures and Tables

**Table 1:** WAXS setup characteristics

Quantity	Value
Wavelength (Å)	1.04
Lowest attainable *q* (*q*_min_) (Å^−1^)	0.06
Highest attainable *q* (*q*_max_) (Å^−1^)	2.2
Dynamic angular range (2θ) (°)	3.3–21.2
Typical beam-stop size (mm)	0.5
Lowest possible sample-to-detector distance (mm)	207.5
Highest possible sample-to-detector distance (mm)	357.0
X-ray beam size (horizontal × vertical) (mm^2^)	0.3 × 0.3
X-ray beam flux (photons s^−1^)	∼10^11^

**Table 2:** Experimental details

Experiment	Measurement	Sample concentration, volume used	Exposure per frame (s), on sample (s)†	Temperature (K)	Flow rate (µl s^−1^)
Lanthanum hexaborate for distance calibration	Single 2D powder diffraction image	LaB_6_ salt in 1 mm capillary	5, NA	298	NA
Light-induced conformational change in phytochromes	Difference scattering due to exposure to 780 and 660 nm light	14 mg ml^−1^, 400 µl	12, 0.5	298	0.5
Ca^2+^-induced structural change in SoPIP2;1	Difference scattering due to UV-laser-induced release of Ca^2+^	15 mg ml^−1^, 300 µl	30, 4.7	283	0.05

† This value corresponds to the time required for the sample to flow 300 µm through the X-ray beam.

## References

[bb1] Ahn, S., Kim, K. H., Kim, Y., Kim, J. & Ihee, H. (2009). *J. Phys. Chem. B*, **113**, 13131–13133. 10.1021/jp906983v 19757799

[bb2] Alleva, K., Niemietz, C. M., Sutka, M., Maurel, C., Parisi, M., Tyerman, S. D. & Amodeo, G. (2006). *J. Exp. Bot.* **57**, 609–621. 10.1093/jxb/erj046 16397000

[bb3] Andersson, M., Malmerberg, E., Westenhoff, S., Katona, G., Cammarata, M., Wöhri, A. B., Johansson, L. C., Ewald, F., Eklund, M., Wulff, M., Davidsson, J. & Neutze, R. (2009). *Structure*, **17**, 1265–1275. 10.1016/j.str.2009.07.007 19748347

[bb4] Arnlund, D., Johansson, L. C., Wickstrand, C., Barty, A., Williams, G. J., Malmerberg, E., Davidsson, J., Milathianaki, D., DePonte, D. P., Shoeman, R. L., Wang, D., James, D., Katona, G., Westenhoff, S., White, T. A., Aquila, A., Bari, S., Berntsen, P., Bogan, M., van Driel, T. B., Doak, R. B., Kjaer, K. S., Frank, M., Fromme, R., Grotjohann, I., Henning, R., Hunter, M. S., Kirian, R. A., Kosheleva, I., Kupitz, C., Liang, M., Martin, A. V., Nielsen, M. M., Messerschmidt, M., Seibert, M. M., Sjöhamn, J., Stellato, F., Weierstall, U., Zatsepin, N. A., Spence, J. C., Fromme, P., Schlichting, I., Boutet, S., Groenhof, G., Chapman, H. N. & Neutze, R. (2014). *Nat. Methods*, **11**, 923–926.

[bb5] Barends, T. R., Foucar, L., Ardevol, A., Nass, K., Aquila, A., Botha, S., Doak, R. B., Falahati, K., Hartmann, E., Hilpert, M., Heinz, M., Hoffmann, M. C., Kofinger, J., Koglin, J. E., Kovacsova, G., Liang, M., Milathianaki, D., Lemke, H. T., Reinstein, J., Roome, C. M., Shoeman, R. L., Williams, G. J., Burghardt, I., Hummer, G., Boutet, S. & Schlichting, I. (2015). *Science*, **350**, 445–450. 10.1126/science.aac5492 26359336

[bb6] Berntsson, O., Diensthuber, R. P., Panman, M. R., Björling, A., Gustavsson, E., Hoernke, M., Hughes, A. J., Henry, L., Niebling, S., Takala, H., Ihalainen, J. A., Newby, G., Kerruth, S., Heberle, J., Liebi, M., Menzel, A., Henning, R., Kosheleva, I., Möglich, A. & Westenhoff, S. (2017). *Nat. Commun.* **8**, 284. 10.1038/s41467-017-00300-5 PMC5561222 28819239

[bb7] Björling, A., Berntsson, O., Takala, H., Gallagher, K. D., Patel, H., Gustavsson, E., **St**, Peter, R., Duong, P., Nugent, A., Zhang, F., Berntsen, P., Appio, R., Rajkovic, I., Lehtivuori, H., Panman, M. R., Hoernke, M., Niebling, S., Harimoorthy, R., Lamparter, T., Stojković, E. A., Ihalainen, J. A. & Westenhoff, S. (2015). *J. Phys. Chem. Lett.* **6**, 3379–3383. 10.1021/acs.jpclett.5b01629 26275765

[bb8] Blanchet, C. E., Zozulya, A. V., Kikhney, A. G., Franke, D., Konarev, P. V., Shang, W., Klaering, R., Robrahn, B., Hermes, C., Cipriani, F., Svergun, D. I. & Roessle, M. (2012). *J. Appl. Cryst.* **45**, 489–495.

[bb9] Cammarata, M., Levantino, M., Schotte, F., Anfinrud, P. A., Ewald, F., Choi, J., Cupane, A., Wulff, M. & Ihee, H. (2008). *Nat. Methods*, **5**, 881–886. 10.1038/nmeth.1255 PMC3159148 18806790

[bb10] Cho, H. S., Dashdorj, N., Schotte, F., Graber, T., Henning, R. & Anfinrud, P. (2010). *Proc. Natl Acad. Sci. USA*, **107**, 7281–7286. 10.1073/pnas.1002951107 PMC2867760 20406909

[bb11] Coquelle, N., Sliwa, M., Woodhouse, J., Schirò, G., Adam, V., Aquila, A., Barends, T. R. M., Boutet, S., Byrdin, M., Carbajo, S., Mora, E. D., Doak, R. B., Feliks, M., Fieschi, F., Foucar, L., Guillon, V., Hilpert, M., Hunter, M. S., Jakobs, S., Koglin, J. E., Kovacsova, G., Lane, T. J., Lévy, B., Liang, M., Nass, K., Ridard, J., Robinson, J. S., Roome, C. M., Ruckebusch, C., Seaberg, M., Thepaut, M., Cammarata, M., Demachy, I., Field, M., Shoeman, R. L., Bourgeois, D., Colletier, J.-P., Schlichting, I. & Weik, M. (2018). *Nat. Chem.* **10**, 31–37.

[bb12] Dubuisson, J.-M., Decamps, T. & Vachette, P. (1997). *J. Appl. Cryst.* **30**, 49–54.

[bb13] Ellis-Davies, G. C., Kaplan, J. H. & Barsotti, R. J. (1996). *Biophys. J.* **70**, 1006–1016. 10.1016/S0006-3495(96)79644-3 PMC1225001 8789118

[bb14] Fischetti, R. F., Rodi, D. J., Gore, D. B. & Makowski, L. (2004). *Chem. Biol.* **11**, 1431–1443. 10.1016/j.chembiol.2004.08.013 15489170

[bb15] Fuller, F. D., Gul, S., Chatterjee, R., Burgie, E. S., Young, I. D., Lebrette, H., Srinivas, V., Brewster, A. S., Michels-Clark, T., Clinger, J. A., Andi, B., Ibrahim, M., Pastor, E., de Lichtenberg, C., Hussein, R., Pollock, C. J., Zhang, M., Stan, C. A., Kroll, T., Fransson, T., Weninger, C., Kubin, M., Aller, P., Lassalle, L., Bräuer, P., Miller, M. D., Amin, M., Koroidov, S., Roessler, C. G., Allaire, M., Sierra, R. G., Docker, P. T., Glownia, J. M., Nelson, S., Koglin, J. E., Zhu, D., Chollet, M., Song, S., Lemke, H., Liang, M., Sokaras, D., Alonso-Mori, R., Zouni, A., Messinger, J., Bergmann, U., Boal, A. K., Bollinger, J. M. Jr, Krebs, C., Högbom, M., Phillips, G. N. Jr, Vierstra, R. D., Sauter, N. K., Orville, A. M., Kern, J., Yachandra, V. K. & Yano, J. (2017). *Nat. Methods*, **14**, 443–449.

[bb16] Hadfield, A. & Hajdu, J. (1993). *J. Appl. Cryst.* **26**, 839–842.

[bb101] Hammersley, A. P. (2016). *J. Appl. Cryst.* **49**, 646–652.

[bb17] Hammersley, A. P., Svensson, S. O. & Thompson, A. (1994). *Nucl. Instrum. Methods Phys. Res. A*, **346**, 312–321.

[bb18] Hirai, M., Koizumi, M., Hayakawa, T., Takahashi, H., Abe, S., Hirai, H., Miura, K. & Inoue, K. (2004). *Biochemistry*, **43**, 9036–9049. 10.1021/bi0499664 15248761

[bb28] Hopkins, J. B., Gillilan, R. E. & Skou, S. (2017). *J. Appl. Cryst.* **50**, 1545–1553. 10.1107/S1600576717011438 PMC5627684 29021737

[bb19] Järvå, M. (2015). PhD thesis, University of Gothenburg, Sweden. https://gupea.ub.gu.se/bitstream/2077/38173/5/gupea_2077_38173_5.pdf.

[bb20] Kaplan, J. H. & Somlyo, A. P. (1989). *Trends Neurosci.* **12**, 54–59. 10.1016/0166-2236(89)90136-7 2469211

[bb21] Levantino, M., Schirò, G., Lemke, H. T., Cottone, G., Glownia, J. M., Zhu, D., Chollet, M., Ihee, H., Cupane, A. & Cammarata, M. (2015). *Nat. Commun.* **6**, 6772. 10.1038/ncomms7772 PMC4396393 25832715

[bb22] Makowski, L. (2010). *J. Struct. Funct. Genomics*, **11**, 9–19. 10.1007/s10969-009-9075-x PMC3057577 20049539

[bb24] Malmerberg, E., Bovee-Geurts, P. H. M., Katona, G., Deupi, X., Arnlund, D., Wickstrand, C., Johansson, L. C., Westenhoff, S., Nazarenko, E., Schertler, G. F., Menzel, A., de Grip, W. J. & Neutze, R. (2015). *Sci. Signal.* **8**, ra26. 10.1126/scisignal.2005646 25759477

[bb23] Malmerberg, E., Omran, Z., Hub, J. S., Li, X., Katona, G., Westenhoff, S., Johansson, L. C., Andersson, M., Cammarata, M., Wulff, M., van der Spoel, D., Davidsson, J., Specht, A. & Neutze, R. (2011). *Biophys. J.* **101**, 1345–1353. 10.1016/j.bpj.2011.07.050 PMC3177065 21943415

[bb25] Minh, D. D. & Makowski, L. (2013). *Biophys. J.* **104**, 873–883. 10.1016/j.bpj.2012.12.019 PMC3576546 23442966

[bb26] Nango, E., Royant, A., Kubo, M., Nakane, T., Wickstrand, C., Kimura, T., Tanaka, T., Tono, K., Song, C., Tanaka, R., Arima, T., Yamashita, A., Kobayashi, J., Hosaka, T., Mizohata, E., Nogly, P., Sugahara, M., Nam, D., Nomura, T., Shimamura, T., Im, D., Fujiwara, T., Yamanaka, Y., Jeon, B., Nishizawa, T., Oda, K., Fukuda, M., Andersson, R., Båth, P., Dods, R., Davidsson, J., Matsuoka, S., Kawatake, S., Murata, M., Nureki, O., Owada, S., Kameshima, T., Hatsui, T., Joti, Y., Schertler, G., Yabashi, M., Bondar, A. N., Standfuss, J., Neutze, R. & Iwata, S. (2016). *Science*, **354**, 1552–1557. 10.1126/science.aah3497 28008064

[bb100] Nielsen, S. S., Toft, K. N., Snakenborg, D., Jeppesen, M. G., Jacobsen, J. K., Vestergaard, B., Kutter, J. P. & Arleth, L. (2009). *J. Appl. Cryst.* **42**, 959–964.

[bb27] Neutze, R. & Moffat, K. (2012). *Curr. Opin. Struct. Biol.* **22**, 651–659. 10.1016/j.sbi.2012.08.006 PMC3520507 23021004

[bb29] Nogly, P., James, D., Wang, D., White, T. A., Zatsepin, N., Shilova, A., Nelson, G., Liu, H., Johansson, L., Heymann, M., Jaeger, K., Metz, M., Wickstrand, C., Wu, W., Båth, P., Berntsen, P., Oberthuer, D., Panneels, V., Cherezov, V., Chapman, H., Schertler, G., Neutze, R., Spence, J., Moraes, I., Burghammer, M., Standfuss, J. & Weierstall, U. (2015). *IUCrJ*, **2**, 168–176. 10.1107/S2052252514026487 PMC4392771 25866654

[bb30] Nogly, P., Weinert, T., James, D., Carbajo, S., Ozerov, D., Furrer, A., Gashi, D., Borin, V., Skopintsev, P., Jaeger, K., Nass, K., Bath, P., Bosman, R., Koglin, J., Seaberg, M., Lane, T., Kekilli, D., Brunle, S., Tanaka, T., Wu, W., Milne, C., White, T., Barty, A., Weierstall, U., Panneels, V., Nango, E., Iwata, S., Hunter, M., Schapiro, I., Schertler, G., Neutze, R. & Standfuss, J. (2018). *Science*, **361**, 6398. 10.1126/science.aat0094 29903883

[bb31] Pande, K., Hutchison, C. D., Groenhof, G., Aquila, A., Robinson, J. S., Tenboer, J., Basu, S., Boutet, S., DePonte, D. P., Liang, M., White, T. A., Zatsepin, N. A., Yefanov, O., Morozov, D., Oberthuer, D., Gati, C., Subramanian, G., James, D., Zhao, Y., Koralek, J., Brayshaw, J., Kupitz, C., Conrad, C., Roy-Chowdhury, S., Coe, J. D., Metz, M., Xavier, P. L., Grant, T. D., Koglin, J. E., Ketawala, G., Fromme, R., rajer, V., Henning, R., Spence, J. C., Ourmazd, A., Schwander, P., Weierstall, U., Frank, M., Fromme, P., Barty, A., Chapman, H. N., Moffat, K., van Thor, J. J. & Schmidt, M. (2016). *Science*, **352**, 725–729.

[bb32] Ramachandran, P. L., Lovett, J. E., Carl, P. J., Cammarata, M., Lee, J. H., Jung, Y. O., Ihee, H., Timmel, C. R. & van Thor, J. J. (2011). *J. Am. Chem. Soc.* **133**, 9395–9404. 10.1021/ja200617t 21627157

[bb33] Rodi, D. J., Mandava, S., Gore, D. B., Makowski, L. & Fischetti, R. F. (2007). *J. Biomol. Screen.* **12**, 994–998. 10.1177/1087057107306104 17942792

[bb34] Suga, M., Akita, F., Sugahara, M., Kubo, M., Nakajima, Y., Nakane, T., Yamashita, K., Umena, Y., Nakabayashi, M., Yamane, T., Nakano, T., Suzuki, M., Masuda, T., Inoue, S., Kimura, T., Nomura, T., Yonekura, S., Yu, L. J., Sakamoto, T., Motomura, T., Chen, J. H., Kato, Y., Noguchi, T., Tono, K., Joti, Y., Kameshima, T., Hatsui, T., Nango, E., Tanaka, R., Naitow, H., Matsuura, Y., Yamashita, A., Yamamoto, M., Nureki, O., Yabashi, M., Ishikawa, T., Iwata, S. & Shen, J. R. (2017). *Nature*, **543**, 131–135.

[bb35] Takala, H., Björling, A., Berntsson, O., Lehtivuori, H., Niebling, S., Hoernke, M., Kosheleva, I., Henning, R., Menzel, A., Ihalainen, J. A. & Westenhoff, S. (2014). *Nature*, **509**, 245–248. 10.1038/nature13310 PMC4015848 24776794

[bb36] Tenboer, J., Basu, S., Zatsepin, N., Pande, K., Milathianaki, D., Frank, M., Hunter, M., Boutet, S., Williams, G. J., Koglin, J. E., Oberthuer, D., Heymann, M., Kupitz, C., Conrad, C., Coe, J., Roy-Chowdhury, S., Weierstall, U., James, D., Wang, D., Grant, T., Barty, A., Yefanov, O., Scales, J., Gati, C., Seuring, C., Srajer, V., Henning, R., Schwander, P., Fromme, R., Ourmazd, A., Moffat, K., Van Thor, J. J., Spence, J. C., Fromme, P., Chapman, H. N. & Schmidt, M. (2014). *Science*, **346**, 1242–1246. 10.1126/science.1259357 PMC4361027 25477465

[bb37] Tiede, D. M., Zhang, R. & Seifert, S. (2002). *Biochemistry*, **41**, 6605–6614. 10.1021/bi015931h 12022864

[bb38] Törnroth-Horsefield, S., Wang, Y., Hedfalk, K., Johanson, U., Karlsson, M., Tajkhorshid, E., Neutze, R. & Kjellbom, P. (2006). *Nature*, **439**, 688–694. 10.1038/nature04316 16340961

[bb39] Ursby, T., Mammen, C. B., Cerenius, Y., Svensson, C., Sommarin, B., Fodje, M. N., Kvick, Å., Logan, D. T., Als-Nielsen, J., Thunnissen, M. M. G. M., Larsen, S. & Liljas, A. (2004). *AIP Conf. Proc.* **705**, 1241–1246.

[bb40] Weierstall, U., James, D., Wang, C., White, T. A., Wang, D., Liu, W., Spence, J. C., Bruce Doak, R., Nelson, G., Fromme, P., Fromme, R., Grotjohann, I., Kupitz, C., Zatsepin, N. A., Liu, H., Basu, S., Wacker, D., Won Han, G., Katritch, V., Boutet, S., Messerschmidt, M., Williams, G. J., Koglin, J. E., Marvin Seibert, M., Klinker, M., Gati, C., Shoeman, R. L., Barty, A., Chapman, H. N., Kirian, R. A., Beyerlein, K. R., Stevens, R. C., Li, D., Shah, S. T., Howe, N., Caffrey, M. & Cherezov, V. (2014). *Nat. Commun.* **5**, 3309. 10.1038/ncomms4309 PMC4061911 24525480

[bb41] Weierstall, U., Spence, J. C. & Doak, R. B. (2012). *Rev. Sci. Instrum.* **83**, 035108. 10.1063/1.3693040 22462961

[bb42] Westenhoff, S., Malmerberg, E., Arnlund, D., Johansson, L., Nazarenko, E., Cammarata, M., Davidsson, J., Chaptal, V., Abramson, J., Katona, G., Menzel, A. & Neutze, R. (2010). *Nat. Methods*, **7**, 775–776. 10.1038/nmeth1010-775c 20885435

